# A simple and rapid method for preparing the whole section of starchy seed to investigate the morphology and distribution of starch in different regions of seed

**DOI:** 10.1186/s13007-018-0283-x

**Published:** 2018-02-21

**Authors:** Lingxiao Zhao, Ting Pan, Dongwei Guo, Cunxu Wei

**Affiliations:** 1 Key Laboratory of Crop Genetics and Physiology of Jiangsu Province/Key Laboratory of Plant Functional Genomics of the Ministry of Education, Yangzhou University, Yangzhou, 225009 China; 2 Co-Innovation Center for Modern Production Technology of Grain Crops of Jiangsu Province/Joint International Research Laboratory of Agriculture & Agri-Product Safety of the Ministry of Education, Yangzhou University, Yangzhou, 225009 China; 3 Maize Biology and Genetic Laboratory in Northwest Arid Area in China, Ministry of Agriculture, Northwest A & F University, Yangling, 712100 China

**Keywords:** Starch granule, Morphology, Distribution, Starchy seed, Whole section of seed

## Abstract

**Background:**

Storage starch in starchy seed influences the seed weight and texture, and determines its applications in food and nonfood industries. Starch granules from different plant sources have significantly different shapes and sizes, and even more the difference exists in the different regions of the same tissue. Therefore, it is very important to in situ investigate the morphology and distribution of starch in the whole seed. However, a simple and rapid method is deficient to prepare the whole section of starchy seed for investigating the morphology and distribution of starch in the whole seeds for a large number of samples.

**Results:**

A simple and rapid method was established to prepare the whole section of starchy seed, especially for floury seed, in this study. The whole seeds of translucent and chalky rice, vitreous and floury maize, and normal barley and wheat were sectioned successfully using the newly established method. The iodine-stained section clearly exhibited the shapes and size of starch granules in different regions of seed. The starch granules with different morphologies and iodine-staining colors existed regionally in the seeds of high-amylose rice and maize. The sections of lotus and kidney bean seeds also showed the feasibility of this method for starchy non-cereal seeds.

**Conclusion:**

The simple and rapid method was proven effective for preparing the whole sections of starchy seeds. The whole section of seed could be used to investigate the morphology and distribution of starch granules in different regions of the whole seed. The method was especially suitable for large sample numbers to investigate the starch morphology in short time.

## Background

Storage starch is synthesized as discrete semicrystalline granules in amyloplast. Cereal starch is the major component of mature seeds, serves as the primary carbohydrate in the diets of humans and livestock, and has numerous important industrial applications [1]. Some legume seeds, such as kidney bean (*Phaseolus vulgaris* Linn.), have high starch content [2]. Lotus seeds are also rich in starch [3]. The starch in starchy seed plant significantly influences the seed weight and texture, and determines its applications in food and nonfood industries [4, 5]. Therefore, the study of seed starch always draws the attention of researchers.

Starch granules from different plant sources have significantly different shapes, sizes, and hilum positions [6]. The differences may be attributed to the biological origin, biochemistry of the amyloplast, and physiology of the plant [7]. The starches from the different organs (such as seed and rhizome) [8] or tissues (such as endosperm and pericarp) of the same plant species have also different granule morphologies [9]. Even more, the lenticular large starch granules and spherical small starch granules coexist in the same endosperm cell of *Triticeae* crops [9–11]. Recently, starch granules with different morphologies or iodine-staining colors (nominated as heterogeneous starch granules) are observed in the endosperm of some cereal high-amylose transgenic or mutant lines [12–14]. These heterogeneous starch granules are regionally distributed in the endosperm, and show significantly different structural and functional properties [12, 13, 15]. In addition, Zhao et al. [16] found that the starch granules in different regions of normal rice and maize endosperm have significantly different sizes. For the above reasons, it is very important to in situ investigate the morphology and distribution of starch granules in the whole seed, especially for the seeds with heterogeneous starch granules.

For in situ observation of starch in seed, the conventional method is to embed the small part of seed in epoxy or spurr resin after chemical fixation [9–11]. This method is capable for preparing semithin section of young endosperm, but it is impossible to obtain the whole section of mature seed. Andersson et al. [17] and Jääskeläinen et al. [18] used the historesin embedding kit to successfully obtain the whole section of barley and wheat mature seeds with 4 μm thickness for investigating seed structure. Recently, Zhao et al. [16] established a method for preparing the whole section of mature cereal seeds with 2 μm thickness to visualize the morphology of endosperm cell and starch and the distribution of starch and protein in whole seed using LR White resin, a low-viscosity and high-permeability resin. However, the chemical fixation, dehydration, resin permeation, and embedding processes of sample are needed before resin sectioning, which takes long time to obtain the section and makes it unsuitable for large sample numbers to investigate the starch morphology [4]. Matsushima et al. [4] reported a rapid method to prepare thin section of cereal mature seed using the razor blade without resin embedding. Compared with resin embedding method, the method is simpler and faster for observation of starch morphology and is highly suitable for the investigation of a large number of samples in short time. However, in fact, it is very difficult to successfully prepare the section. In addition, it is impossible for obtaining the complete section of whole seed, and the uneven section thickness makes observation image vague. Liu et al. [12] and Wellner et al. [19] successfully prepared the section of mature maize seed using glass knife under ultramicrotome instead of razor blade. This method is simple, and it is easy to obtain good section for vitreous maize endosperm. However, the method has difficulty in preparing the whole section of mature seed, and especially is not suitable for floury seed. Some normal crop seeds have high floury kernels, and the mutant and transgenic lines of starch synthesis-related enzymes always exhibit floury seeds [4, 13]. Therefore, it is necessary to establish a simple and rapid method for preparing the whole section of mature seed, especially floury seed in order to in situ investigate the starch granules in a large number of samples.

In this study, our objective was to establish a simple and rapid method to prepare the whole section of mature seeds, especially floury seeds without resin embedding. Using this method, we could rapidly observe the morphology and distribution of starch granules in whole seeds of normal cereal crops, and conveniently investigated the heterogeneous starch granules in high-amylose rice and maize seeds. For example, the mature seed of high-amylose rice TRS shows floury endosperm and has polygonal, aggregate, elongated, and hollow starch granules. In order to investigate the spatial distribution of different morphology granules in endosperm, the whole seed is embedded in LR White resin and sectioned using our previously established method for preparing the whole section of mature cereal seed. The complete section of whole seed clearly exhibits that the polygonal, aggregate, elongated, and hollow granules are regionally distributed in a single seed from inside to outside of the endosperm [13, 16]. However, the preparation of whole section, from fixation, dehydration, permeation to embedment, takes over one week. If using the newly established method, the preparation of whole section of seed with high quality only took about one hour. In addition, the seeds of lotus and kidney bean were also used to confirm the feasibility of this method for preparing the section of starchy non-cereal seeds.

## Methods

### Plant materials

**Fig. 1:**
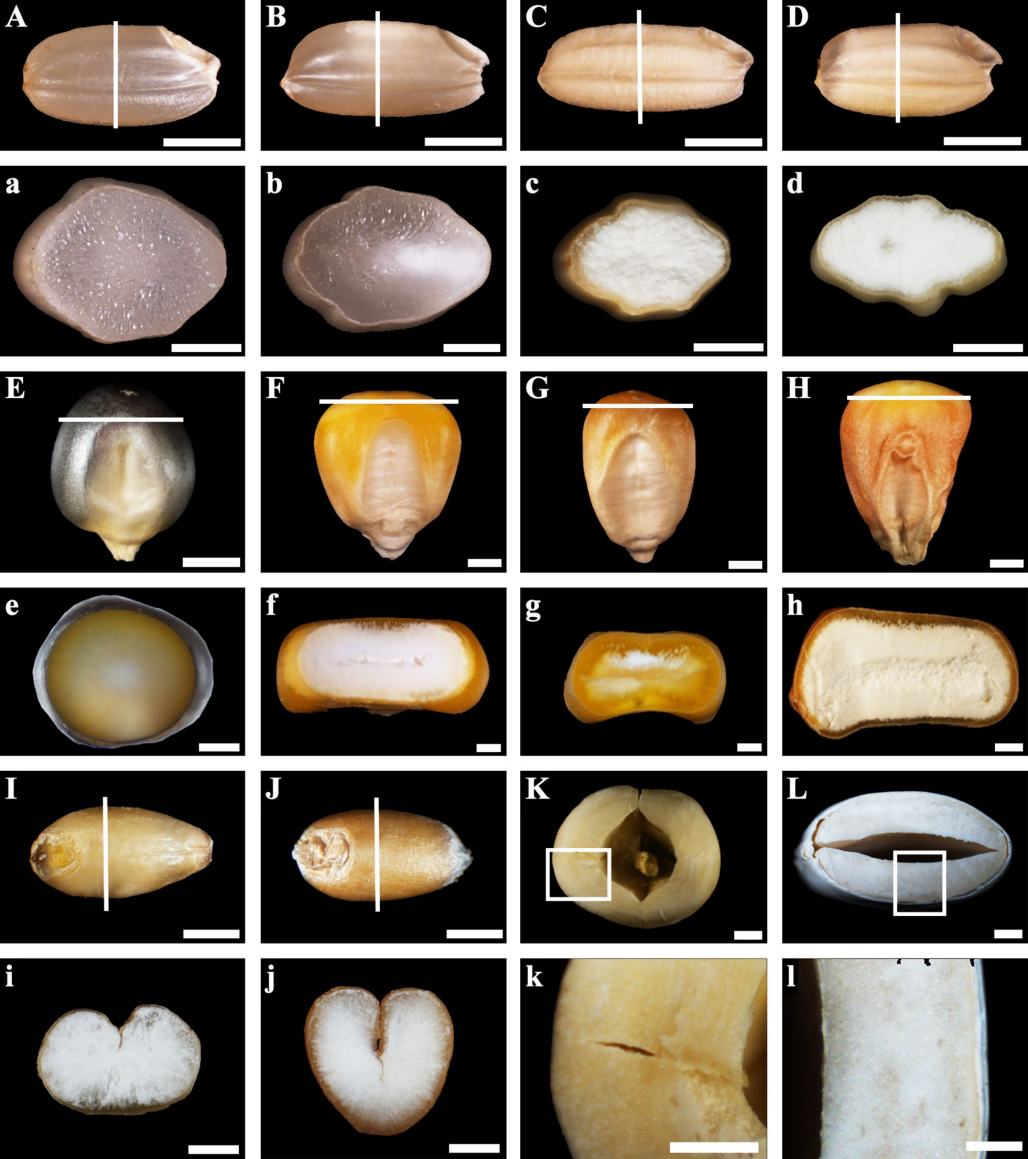
Images of starchy seeds. **A**, **a** normal rice Zhonghua 11; **B**, **b** normal rice Teqing; **C**, **c** high-amylose rice TRS derived from Teqing with inhibiting starch branching enzyme I/IIb; **D**, **d** rice *ae* mutant from Zhonghua 11; **E**, **e** popcorn SK; **F**, **f** normal maize Xianyu 335; **G**, **g** maize *ae* mutant Zae 35; **H**, **h** maize *ae* mutant Zae 50; **I**, **i** normal barley Yangnongpi 5; **J**, **j** normal wheat Yangmai 13; **K**, **k** lotus; **L**, **l** kidney bean. The white line in (**A**–**J**) indicated the section position of (**a**–**j**) and the white frame in (**K**, **L**) indicated the section region of (**k**, **l**). Scale bars = 2 mm for (**A**–**L**) and 1 mm for (**a**–**l**)

Some cereal and non-cereal mature dry seeds were used in this study (Table 1). These cereal crops were grown in the experiment field of Yangzhou University, Yangzhou, China. Lotus and kidney bean seeds were bought from a local natural food market (Yangzhou City, China). The phenotypes and sectioning position of these seeds are shown in Fig. 1.

**Table 1:** Seed samples used in this study

Categories	Species
Cereal seed	*Japonica* rice (*Oryza sativa* L.) cultivar Zhonghua 11 and its *ae* mutant
	*Indica* rice (*Oryza sativa* L.) cultivar Teqing and its transgenic line TRS with inhibition of starch branching enzyme [13]
	Maize (*Zea mays* L.) dent corn inbred line Xianyu 335 and its *ae* mutant Zae35 and Zae50
	Maize (*Zea mays* L.) popcorn inbred line SK
	Barley (*Hordeum vulgare* L.) cultivar Yangnongpi 5
	Wheat (*Triticum aestivum* L.) cultivar Yangmai 13
Non-cereal seed	Lotus (*Nelumbo nucifera* Gaertn.)
	Kidney bean (*Phaseolus vulgaris* Linn.)

### Preparing the whole section of dry seed

**Fig. 2:**
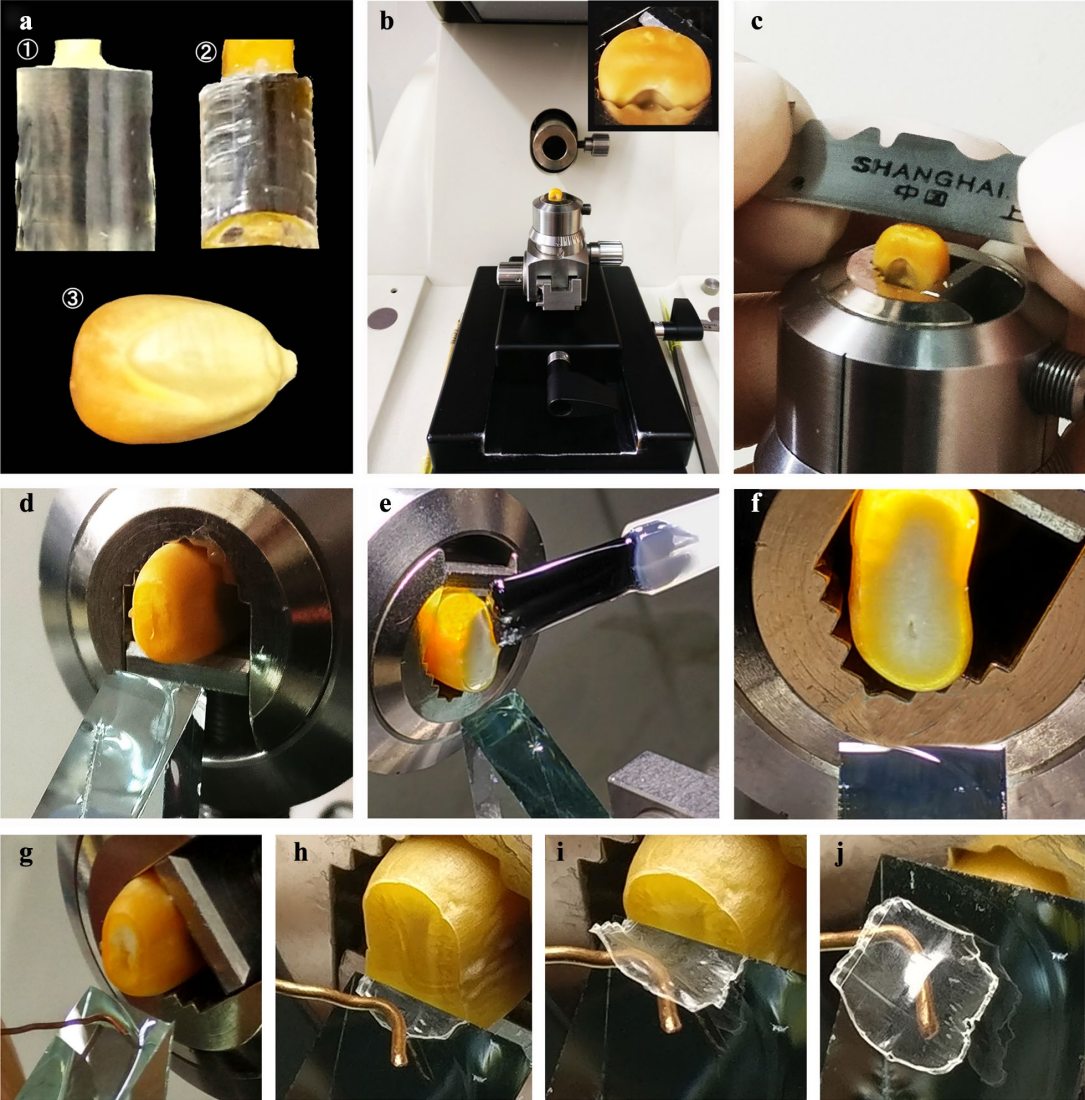
The preparing method of whole section of starchy seed. **a**, **b** the small seeds and the pieces of large seeds were glued to resin blocks (**a**-①, **a**-②), and the resin blocks and some large seeds (**a**-③) were clamped in the trimmer (**a**). **c** Sample surface was trimmed using a razor blade. **d** The surface was polished by a glass knife. **e** For floury seeds, the polished surface of sample was covered with transparent nail polish. **f** The excess nail polish was removed from sample surface using glass knife after nail polish solidification. **g** The hook was put near the blade edge before cutting. **h**, **i** A semithin section of 2 μm thickness was sectioned with the hook preventing section curling up or down. **j** The section was transferred to the slide with the hook

**Fig. 3:**
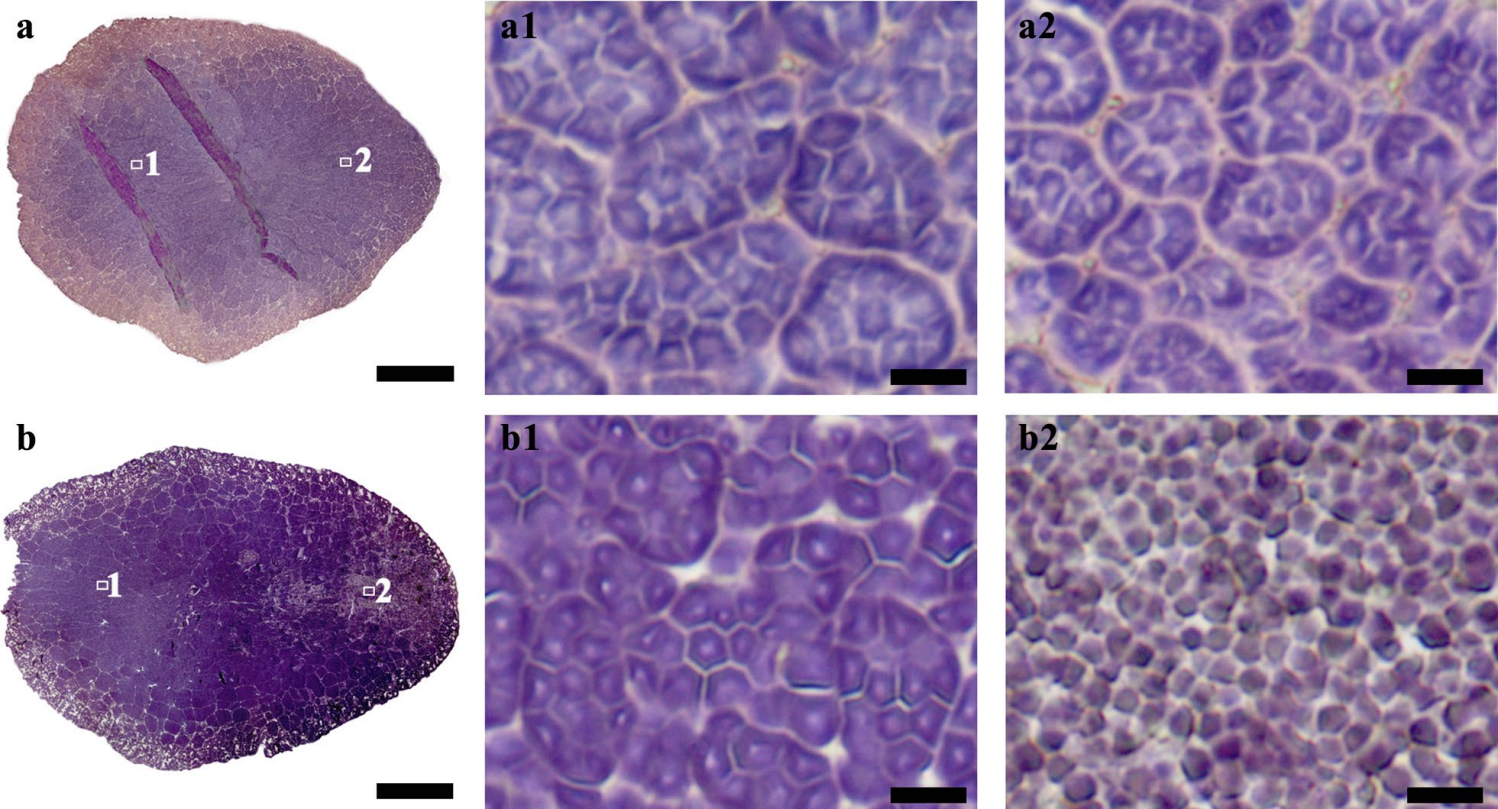
Whole section of normal rice seeds and the morphology and distribution of starch granule. **a** and **b** Whole cross section of normal rice Zhonghua 11 (**a**) and Teqing (**b**). (**a1**–**b2**) Iodine stained starch granules in regions of (**a**, **b**). Scale bars = 500 μm for (**a**, **b**) and 10 μm for (**a1**–**b2**)

The whole section of dry seed was prepared according to the methods of Zhao et al. [16] and Wellner et al. [19] with some major modifications. The detail process is presented in Fig. 2. First, some resin blocks were prepared, and the small-sized seeds including rice and barley seeds were directly glued together with the resin block (Fig. 2a-①). For large-sized seeds such as lotus and kidney bean, the seed was trimmed into small pieces and then glued together with resin block (Fig. 2a-②). The large seed, such as maize seed (Fig. 2a-③) or the resin block with sample was clamped in the block trimmer of Leica ultramicrotome (Fig. 2b). A fresh razor blade was used to trim the top surface of the sample and made it as flat as possible (Fig. 2c). The flat surface was then polished by a glass knife to get mirror surface (Fig. 2d). For floury/chalky seed, one or two drops of transparent nail polish (Temix Professional Lacquer Nail Polish, China) were added onto the polished surface and covered it with the help of brushing hair (Fig. 2e). In order to avoid destroying the sample surface, the brushing hair did not touch it. For safety, the nail polish should be used following manufacturer’s recommendations regarding adequate ventilation. After solidification of nail polish for about 20 min, the surface was further polished with glass knife to remove excess nail polish (Fig. 2f). For transparent/vitreous seed, the treatment of nail polish was omitted. The polished sample was sectioned using glass knife, and a small copper hook was put closely to the knife edge before cutting (Fig. 2g). The hook could prevent the semithin section curling upward (Fig. 2h) and adhering to the glass knife (Fig. 2i) during section cutting. Finally, the section with 2 μm thickness was carefully transferred with the hook to a small drop of distilled water on a glass slide (Fig. 2j), and dried on a heating stage for 2 h at 48 °C.

In order to obtain the high quality section of seed, especially for floury seed, some details needed to be concerned by operator. Firstly, brand-new and sharp razor blades and glass knives were used to trim and section the sample. Secondly, the nail polish should be completely solidified before sectioning. Third, the sectioning speed should be slow enough to avoid destroying the sample. Finally, after treatment of nail polish, the beginning six sections was high quality for floury seeds of maize and rice, and only two or three sections could be used to observe the morphology for floury seeds of wheat, barley, lotus and kidney bean.

### Staining and observation of section

The semithin sections were stained in 20 μL of iodine solution (25% glycerol, 0.07% I_2_ and 0.14% KI) for 5 min in darkness, and covered with coverslip. The starch granules were observed and photographed using an Olympus BX53 light microscope equipped with a CCD camera. For birefringence observation, the sections immersed with 20 μL of 25% glycerol were viewed and photographed under polarized light.

## Results and discussion

In the present study, a simple and rapid sectioning method was established to prepare the whole section of starchy cereal seeds and sample pieces of large starchy non-cereal seeds (Fig. 2). This section could be used to in situ investigate the morphology and distribution of starch granules in the whole seeds.

### The whole section of normal rice seeds and the morphology and distribution of starch granule

Normal rice endosperm is commonly divided into two types, i.e. translucent and chalky endosperm depending on chalkiness percentage [20]. Starch granules are packed compactly in translucent endosperm, and loosely in chalky endosperm [21]. *Japonica* rice cultivar Zhonghua 11 and *indica* rice cultivar Teqing were used in this study. Zhonghua 11 seed was translucent (Fig. 1A, a), and could be directly sectioned. Teqing seed had translucent and chalky endosperm (Fig. 1B, b), and needed the treatment of nail polish before sectioning. The whole sections of Zhonghua 11 and Teqing seeds were stained with iodine solution, and are shown in Fig. 3a, b, respectively.

The whole section of seed provided the possibility of investigating the morphology and distribution of starch granule in every region of the seed. For Zhonghua 11 seed, typical compound starches were compactly distributed in every region of the translucent endosperm. The compound starch was consisted of many polygonal subgranules (Fig. 3-a1, a2). These subgranules, each with a central hilum, are individually initiated and developed in an amyloplast during endosperm development [22]. For Teqing seed, the starches in the regions of translucent endosperm were similar to that of Zhonghua 11 in morphology and distribution (Fig. 3-b1). However, the starches in the regions of chalky endosperm showed significantly difference. Polygonal and spherical granules were loosely distributed in ventral endosperm cell with air space between them, and typical compound starches were not observed (Fig. 3-b2). These findings were in accordance with previous literatures that compound starch granules in chalky endosperm were easily broken and released subgranules under external pressure during maturation and drying [23, 24]. Therefore, the existence of numerous air spaces between the loosely packed starch granules resulted in a change in light refraction, which was associated directly with chalky appearance [24, 25].

### The whole section of normal maize seeds and the morphology and distribution of starch granule

**Fig. 4:**
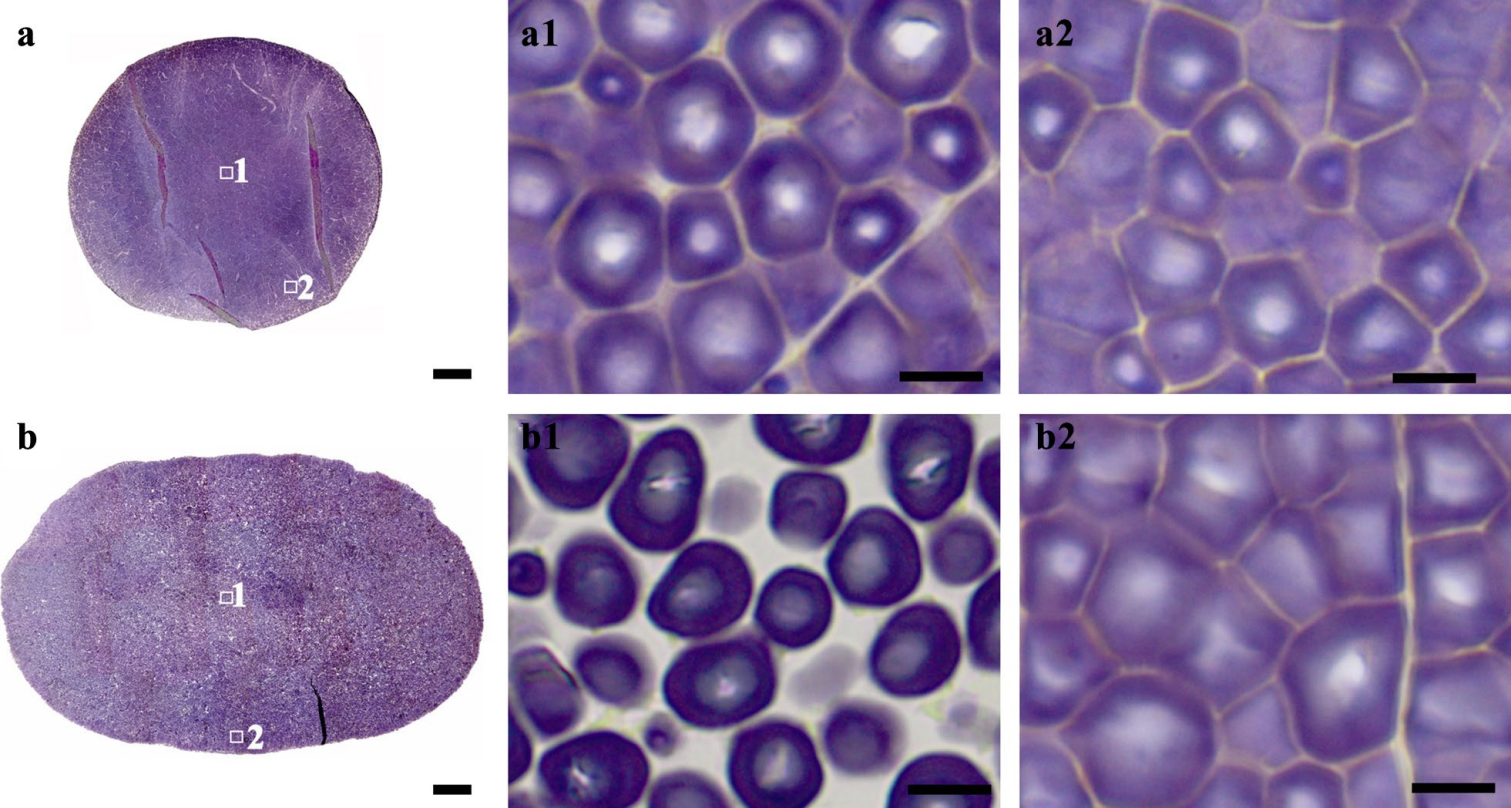
Whole section of normal maize seeds and the morphology and distribution of starch granule. **a**, **b** Whole cross section of normal maize popcorn SK (**a**) and Xianyu 335 (**b**). **a1**–**b2** Iodine stained starch granules in regions of (**a**, **b**). Scale bars = 500 μm for (**a**, **b**) and 10 μm for (**a1**–**b2**)

Maize endosperm is normally classified as vitreous and floury endosperm [26]. Starch granules are packed densely in vitreous endosperm and loosely in floury endosperm [27]. Two normal maize varieties popcorn SK and Xianyu 335 were used in this study. Popcorn SK seed was vitreous (Fig. 1E, e), and could be directly sectioned. Xianyu 335 seed had large floury endosperm (Fig. 1F, f), needed the treatment of nail polish before sectioning. The whole sections of popcorn SK and Xianyu 335 seeds were stained with iodine solution, and are shown in Fig. 4a, b, respectively. For popcorn SK seed, endosperm was compactly filled with polygonal granules, while starch granules in periphery region were significantly smaller than those in center region (Fig. 4-a1, a2). For Xianyu 335 seed, starch granules in central floury endosperm were loosely packed, but those in periphery vitreous endosperm were compactly packed. The granules in central endosperm were spherical, and smaller than those in periphery endosperm, which were polygonal (Fig. 4-b1, b2; [28]).

Starch granules in vitreous and floury endosperm are normally simple granules [29]. Moreover, the formation of vitreous and floury endosperm has been discussed extensively. Starch granules in vitreous endosperm are enclosed in a thin layer of protein. During maturation and drying process, protein is dehydrated and shrinks, pulling starch granules closer. However, water in starch granules makes them flexible. Therefore, starch granules turn compact and polygonal. For floury endosperm, the bond between protein and starch is much weaker and rupture during drying, resulting in air spaces and round starch granules [30]. The loose structure and intergranular air spaces also explain the low density of floury endosperm and cause the opaque appearance by refracting light [30].

### The whole section of normal barley and wheat seeds and the morphology and distribution of starch granule

Starch granules in *Triticeae* endosperm are commonly divided into two categories: large lenticular A-type and small spherical B-type starch granules, between which differences are shown in granular morphology, molecular structure and other chemical properties [31, 32]. Moreover, the distribution of large and small starch granules in wheat and barley endosperm is also detected using resin section method [18]. Therefore, the variation in number ratio of large and small starch granules and their distribution in seed affect grain quality and crop yield.

**Fig. 5:**
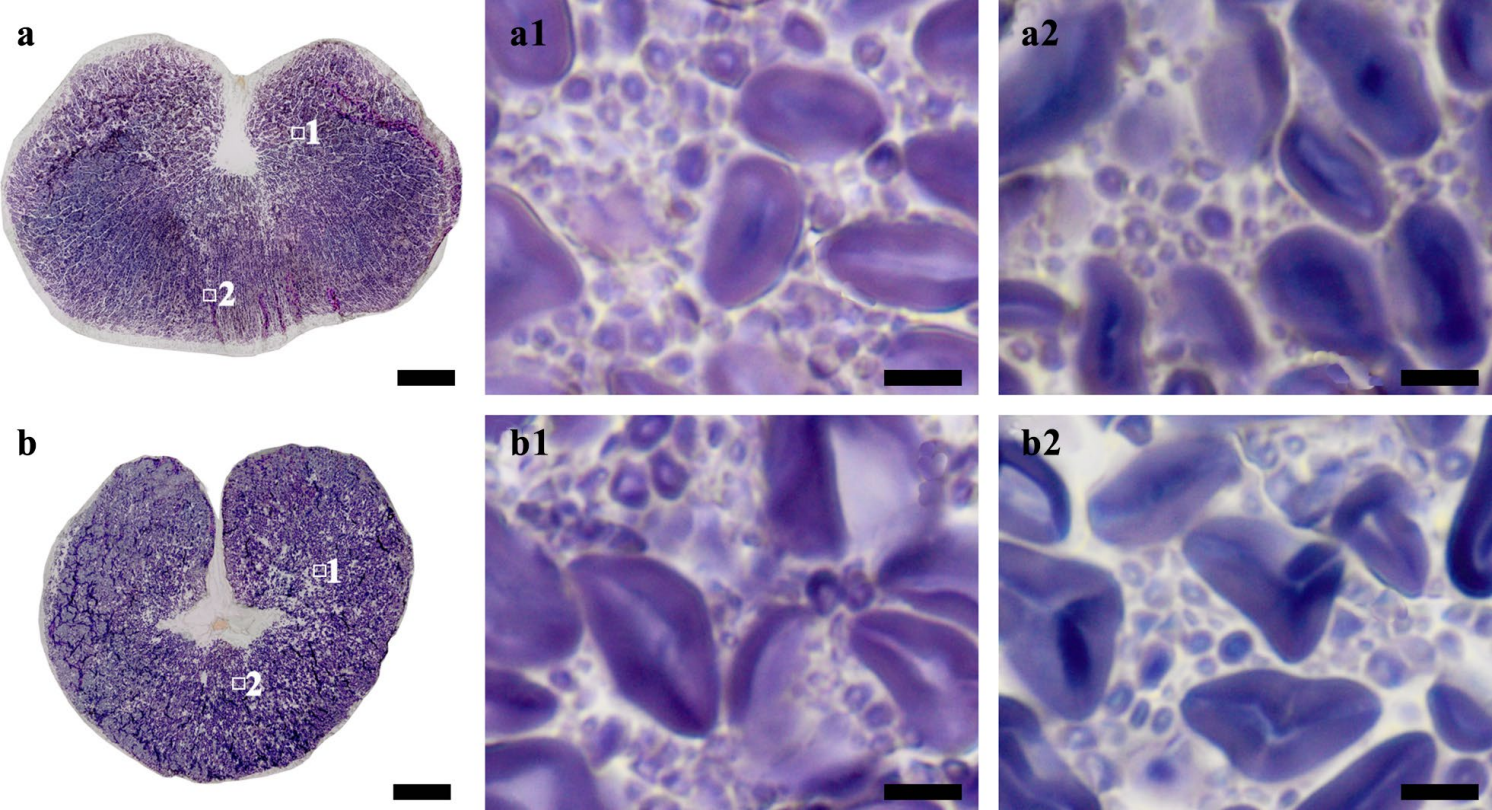
Whole section of normal barley and wheat seeds and the morphology and distribution of starch granule. **a**, **b** Whole cross section of normal barley Yangnongi 5 (**a**) and wheat Yangmai 13 (**b**). **a1**–**b2** Iodine stained starch granules in regions of (**a**, **b**). Scale bars = 500 μm for (**a**, **b**) and 10 μm for (**a1**–**b2**)

In the present study, normal barley variety Yangnongpi 5 and wheat variety Yangmai 13 were adopted to observe and compare the distribution and morphology of starch granules in barley and wheat endosperm. Both samples had soft endosperm (Fig. 1I, i, J, j) and needed treatment of nail polish before sectioning. Stained with iodine solution, whole sections of normal barley and wheat are shown in Fig. 5a, b, respectively. Both large and small starch granules were observed clearly in the dorsal and ventral regions (Fig. 5-a1–b2). More small starch granules existed in the ventral region than the dorsal region. Large starch granules were oval and lenticular and small granules were more spherical, as discussed earlier [18].

### The morphology and distribution of heterogeneous starch granules in high-amylose rice and maize seeds

**Fig. 6:**
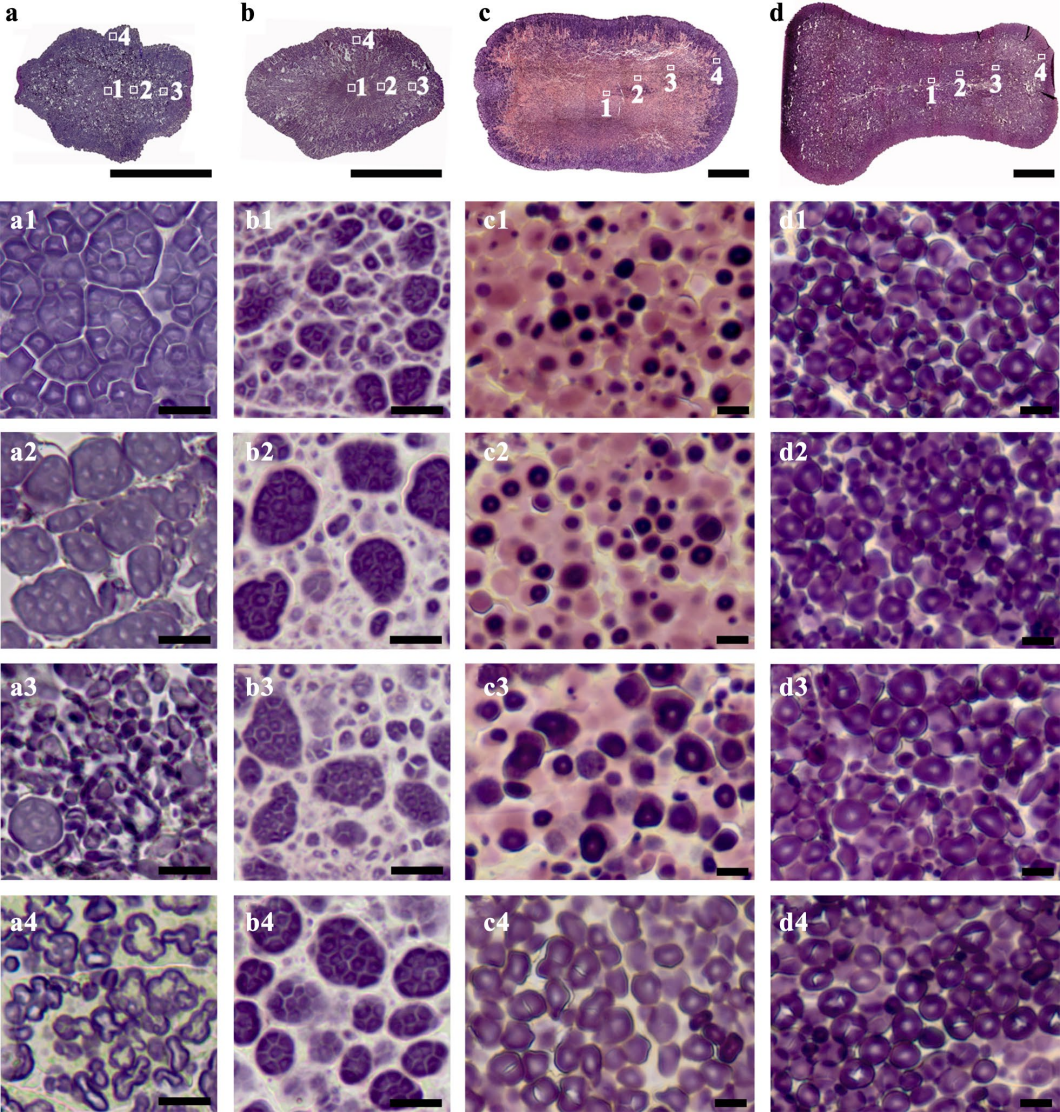
Morphology and distribution of heterogeneous starch granules in high-amylose rice and maize seeds. **a**–**d** Whole cross section of rice TRS (**a**), rice *ae* mutant (**a**), maize *ae* mutant Zae 35 (**C**), and maize *ae* mutant Zae 50 (**d**). **a1**–**d4** Iodine stained starch granules in regions of (**a**–**d**). Scale bars = 1 mm for (**a**–**d**) and 10 μm (**a1**–**d4**)

Starch granules in high-amylose cereal endosperm with mutation or inhibiting expression of starch branching enzyme genes show significant heterogeneity in granule morphology, regional distribution in seed, and starch properties [4, 12–15, 19, 33, 34]. Therefore, it is important to visualize the morphology and distribution of starch granule in whole section of high-amylose cereal seeds. However, the seeds of high-amylose cereal crops are usually floury, and as a consequence whole sections of seeds cannot be obtained using the methods of Matsushima et al. [4] and Liu et al. [12]. In the present study, high-amylose rice and maize seeds were investigated for the morphology and distribution of heterogeneous starch granules in whole seeds. Rice TRS seed was totally floury in the whole endosperm (Fig. 1C, c), rice *ae* mutant seed was mostly floury with a translucent core (Fig. 1D, d), maize Zae 35 was mostly vitreous in top region of the seed (Fig. 1G, g), and maize Zae 50 was totally floury (Fig. 1H, h). For rice TRS, rice *ae* mutant, and maize Zae 50, the floury seeds needed the treatment of nail polish before sectioning, and their whole sections were successfully obtained (Fig. 6a, b, d). For maize Zae 35, the vitreous seed could be directly sectioned to obtain the whole section with thickness of 2 μm (Fig. 6c).

Rice TRS endosperm contains polygonal, aggregate, elongated, and hollow starch granules. The resin section of mature seed shows that they are distributed regionally from the inner to the outer of endosperm [13]. In the present study, the whole section of TRS seed had the similar results with those of resin section (Fig. 6-a1–a4; [13]), indicating that the simple and rapid section method could allow us to investigate starch heterogeneity in whole seed rapidly and easily. Therefore, we rapidly visualized the starch heterogeneity in rice *ae* mutant and maize Zae 35 and Zae 50 seeds. For rice *ae* mutant, compound starch granules were loosely distributed and became larger from center to periphery region, surrounded by smaller starch granules (Fig. 6-b1–b4). Compound starch granules with pink-stained region were distributed in interior region (Fig. 6-b1–b3), while elongated starch granules were observed only in the central endosperm (Fig. 6-b1). For maize Zae 35, biphasic starch granules with pink-stained periphery and dark-stained core were in the majority at the section region (Fig. 6c). These biphasic granules were elucidated sufficiently in literatures about the morphological feature, distribution and structural variability [12, 19]. They were mainly distributed in the region where starch is first deposited during kernel development, which was consistent with the observation results in our study. The pink-stained phenomenon in *japonica* rice and maize *ae* mutant lines was highlighted by Wellner et al. [19] and Liu et al. [12] that might result from the defect of starch branching enzymes during growth. Moreover, the dark-stained core in these biphasic granules were becoming larger and larger from the inner to the outer (Fig. 6-c1–c3), and only oval granules were observed in periphery endosperm (Fig. 6-c4). For maize Zae 50, which had significantly high amylose content [35], overall starch granules were much smaller comparing with Zae 35 (Fig. 6-c1–c4) or normal maize Xianyu 335 (Fig. 4-b1, b2). The smallest and most elongated starch granules were detected in central regions (Fig. 6-d1, d2). Middle and periphery regions contained larger and spherical starch granules and almost no elongated ones (Fig. 6-d3, d4).

### The morphology and distribution of starch granule in starchy non-cereal seed

**Fig. 7:**
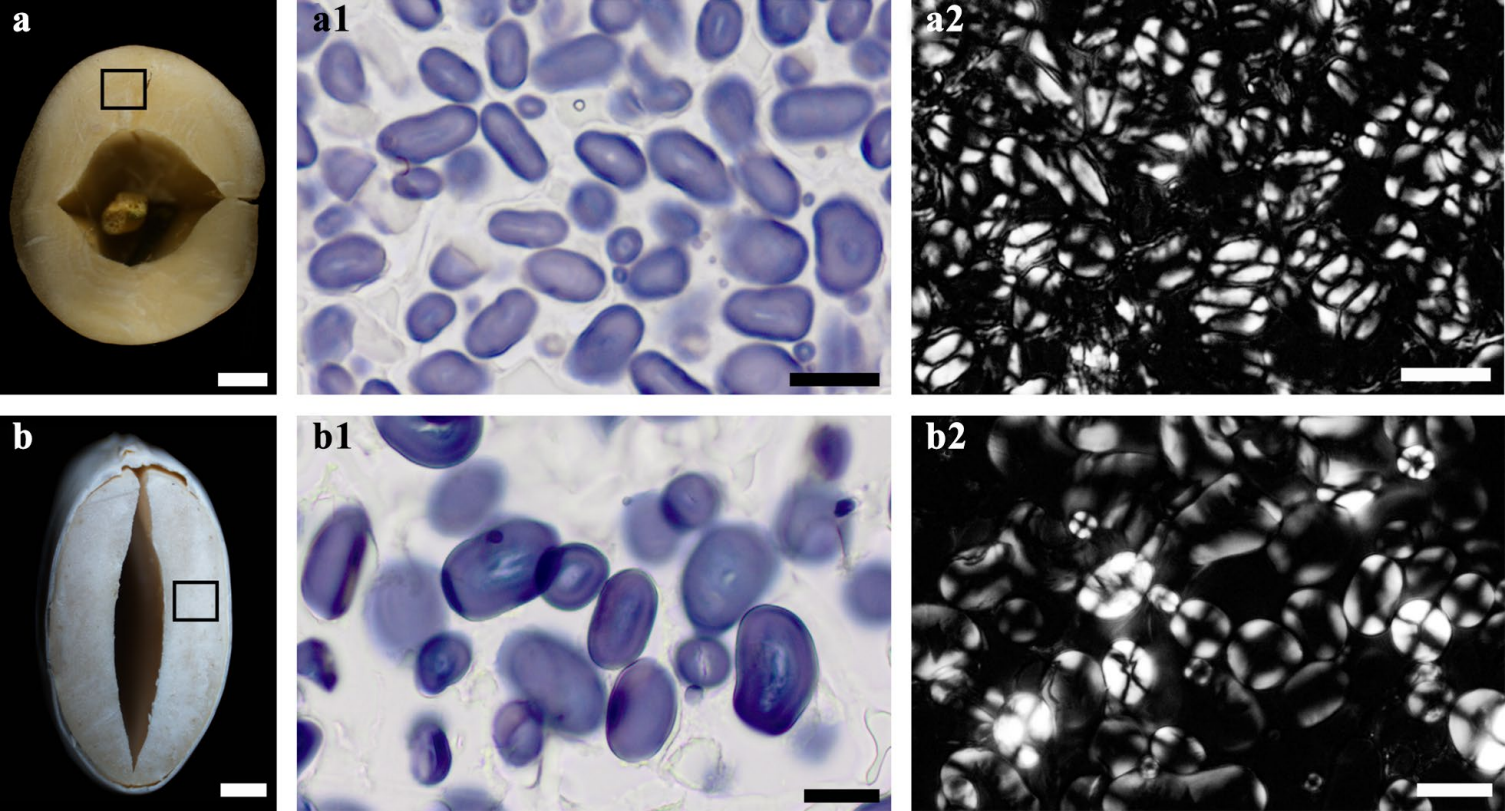
Morphology of starch granule in starchy non-cereal seed. **a**, **b** Sample pieces from lotus (**a**) and kidney bean (**b**) seed. **a1**, **b1**) Iodine stained starch granules in magnified regions of (**a**, **b**). **a2**, **b2** Polarized images of starch granules in magnified regions of (**a**, **b**). Scale bars = 2 mm for (**a**, **b**) and 20 μm for (**a1**–**b2**)

Unlike monocotyledon, dicotyledon stores nutrients in their cotyledons [36]. Here, we selected lotus and kidney bean seeds, which have high starch content in cotyledon [8, 37], to justify the feasibility of the rapid method for preparing the section of starchy non-cereal seeds to observe the starch granules. After treatment of nail polish, the cotyledon could be sectioned successfully and rapidly (Fig. 7a, b). Starch granules in lotus cotyledon were oval and round in shape and different in size (Fig. 7-a1). For kidney bean seed, starch granules exhibiting similar shape were larger than those in lotus cotyledon but packed more loosely (Fig. 7-b1). Under polarized light, unstained starch granules showed birefringence in the form of the typical maltese crosses (Fig. 7-a2, b2), indicating a symmetrical radial molecular orientation in these granules [38].

## Conclusion

A simple and rapid method was established to prepare the whole section of starchy seed. Using this method, the translucent rice seed and vitreous maize seed were directly sectioned, but chalky rice seed, floury maize seed, soft barley and wheat seeds, and starchy non-cereal seed needed the treatment of nail polish before sectioning. The whole section provided the possibility of rapidly investigating the morphology and distribution of starch granules in the whole seed. More importantly, the method could effectively observe the starch heterogeneity of high-amylose cereal crops and was suitable for viewing starch of a large number of cereal transgenic lines or mutants well in short time.

## References

[1] James MG, Denyer K, Myers AM (2003). Starch synthesis in the cereal endosperm. Curr Opin Plant Biol.

[2] Wani IA, Sogi DS, Wani AA, Gill BS, Shivhare US (2010). Physico-chemical properties of starches from Indian kidney bean (*Phaseolus vulgaris*) cultivars. Int J Food Sci Technol.

[3] Guo Z, Zeng S, Lu X, Zhou M, Zheng M, Zheng B (2015). Structural and physicochemical properties of lotus seed starch treated with ultra-high pressure. Food Chem.

[4] Matsushima R, Maekawa M, Fujita N, Sakamoto W (2010). A rapid, direct observation method to isolate mutants with defects in starch grain morphology in rice. Plant Cell Physiol.

[5] Zhang Z, Zheng X, Yang J, Messing J, Wu Y (2016). Maize endosperm-specific transcription factors O2 and PBF network the regulation of protein and starch synthesis. Proc Natl Acad Sci USA.

[6] Jane JL, Kasemsuwan T, Leas S, Zobel H, Robyt JF (1994). Anthology of starch granule morphology by scanning electron microscopy. Starch.

[7] Sandhu KS, Singh N, Kaur M (2004). Characteristics of the different corn types and their grain fractions: physicochemical, thermal, morphological and rheological properties of starches. J Food Eng.

[8] Man J, Cai J, Cai C, Xu B, Huai H, Wei C (2012). Comparison of physicochemical properties of starches from seed and rhizome of lotus. Carbohydr Polym.

[9] Yu X, Zhou L, Zhang J, Yu H, Xiong F, Wang Z (2015). Comparison of starch granule development and physicochemical properties of starches in wheat pericarp and endosperm. J Sci Food Agric.

[10] Wei C, Zhang J, Zhou W, Chen Y, Xu R (2008). Development of small starch granule in barley endosperm. Acta Agron Sin.

[11] Wei C, Zhang J, Chen Y, Zhou W, Xu B, Wang Y, Chen J (2010). Physicochemical properties and development of wheat large and small starch granules during endosperm development. Acta Physiol Plant.

[12] Liu D, Parker ML, Wellner N, Kerby AR, Cross K, Morris VJ, Cheng F (2013). Structural variability between starch granules in wild type and in ae high-amylose mutant maize kernels. Carbohydr Polym.

[13] Cai C, Huang J, Zhao L, Liu Q, Zhang C, Wei C (2014). Heterogeneous structure and spatial distribution in endosperm of high-amylose rice starch granules with different morphologies. J Agric Food Chem.

[14] Jiang H, Horner HT, Pepper TM, Blanco M, Campbell M, Jane JL (2010). Formation of elongated starch granules in high-amylose maize. Carbohydr Polym.

[15] Man J, Lin L, Wang Z, Wang Y, Liu Q, Wei C (2014). Different structure of heterogeneous starch granules from high-amylose rice. J Agric Food Chem.

[16] Zhao L, Pan T, Cai C, Wang J, Wei C (2016). Application of whole sections of mature cereal seeds to visualize the morphology of endosperm cell and starch and the distribution of storage protein. J Cereal Sci.

[17] Andersson AAM, Andersson R, Autio K, Åman P (1999). Chemical composition and microstructure of two naked waxy barleys. J Cereal Sci.

[18] Jääskeläinen AS, Holopainen-Mantila U, Tamminen T, Vuorinen T (2013). Endosperm and aleurone cell structure in barley and wheat as studied by optical and Raman microscopy. J Cereal Sci.

[19] Wellner N, Georget DMR, Parker ML, Morris VJ (2011). In situ Raman microscopy of starch granule structures in wild type and ae mutant maize kernels. Starch.

[20] Li Y, Fan C, Xing Y, Yun P, Luo L, Yan B, Peng B, Xie W, Wang G, Li X, Xiao J, Xu C, He Y (2014). *Chalk5* encodes a vacuolar H^+^-translocating pyrophosphatase influencing grain chalkiness in rice. Nat Genet.

[21] Lin Z, Zheng D, Zhang X, Wang Z, Lei J, Liu Z, Li G, Wang S, Ding Y (2016). Chalky part differs in chemical composition from translucent part of japonica rice grains as revealed by a notched-belly mutant with white-belly. J Sci Food Agric.

[22] Wei C, Qin F, Zhou W, Chen Y, Xu B, Wang Y, Gu M, Liu Q (2010). Formation of semi-compound C-type starch granule in high-amylose rice developed by antisense RNA inhibition of starch-branching enzyme. J Agric Food Chem.

[23] Tashiro T, Wardlaw IF (1991). The effect of high temperature on kernel dimensions and the type and occurrence of kernel damage in rice. Aust J Agric Res.

[24] Xi M, Lin Z, Zhang X, Liu Z, Li G, Wang Q, Wang S, Ding Y (2014). Endosperm structure of white-belly and white-core rice grains shown by scanning electron microscopy. Plant Prod Sci.

[25] Yamakawa H, Hirose T, Kuroda M, Yamaguchi T (2007). Comprehensive expression profiling of rice grain filling-related genes under high temperature using DNA microarray. Plant Physiol.

[26] Wu Y, Holding DR, Messing J (2010). γ-Zeins are essential for endosperm modification in quality protein maize. Proc Natl Acad Sci USA.

[27] Shapter FM, Henry RJ, Lee LS (2008). Endosperm and starch granule morphology in wild cereal relatives. Plant Genet Resour.

[28] Srichuwong S, Curti D, Austin S, King R, Lamothe L, Gloria-Hernandez H (2017). Physicochemical properties and starch digestibility of whole grain sorghums, millet, quinoa and amaranth flours, as affected by starch and non-starch constituents. Food Chem.

[29] Tateoka T (1962). Starch grains of endosperm in grass systematics. Bot Mag Tokyo.

[30] Robutti JL, Hoseney RC, Wassom CE (1974). Modified *opaque*-*2* corn endosperms. II. Structure viewed with a scanning electron microscope. Cereal Chem.

[31] Naguleswaran S, Li J, Vasanthan T, Bressler D, Hoover R (2012). Amylolysis of large and small granules of native triticale, wheat and corn starches using a mixture of α-amylase and glucoamylase. Carbohydr Polym.

[32] Naguleswaran S, Vasanthan T, Hoover R, Bressler D (2013). The susceptibility of large and small granules of waxy, normal and high-amylose genotypes of barley and corn starches toward amylolysis at sub-gelatinization temperatures. Food Res Int.

[33] Cai C, Lin L, Man J, Zhao L, Wang Z, Wei C (2014). Different structural properties of high-amylose maize starch fractions varying in granule size. J Agric Food Chem.

[34] Lin L, Cai C, Gilbert RG, Li E, Wang J, Wei C (2016). Relationships between amylopectin molecular structures and functional properties of different-sized fractions of normal and high-amylose maize starches. Food Hydrocoll.

[35] Lin L, Guo D, Zhao L, Zhang X, Wang J, Zhang F, Wei C (2016). Comparative structure of starches from high-amylose maize inbred lines and their hybrids. Food Hydrocoll.

[36] Toyooka K, Okamoto T, Minamikawa T (2001). Cotyledon cells of *Vigna mungo* seedlings use at least two distinct autophagic machineries for degradation of starch granules and cellular components. J Cell Biol.

[37] Güzel D, Sayar S (2012). Effect of cooking methods on selected physicochemical and nutritional properties of barlotto bean, chickpea, faba bean, and white kidney bean. J Food Sci Technol.

[38] Qin F, Man J, Cai C, Xu B, Gu M, Zhu L, Shi YC, Liu Q, Wei C (2012). Physicochemical properties of high-amylose rice starches during kernel development. Carbohydr Polym.

